# Walking the tightrope-perspectives on local politicians’ role in implementing a national social care policy on evidence-based practice

**DOI:** 10.1186/s13033-016-0107-1

**Published:** 2016-12-19

**Authors:** A. Bäck, C. Ståhl, U. von Thiele Schwarz, A. Richter, H. Hasson

**Affiliations:** 1 Department of Learning, Informatics, Management and Ethics, Medical Management Centre, Karolinska Institutet, 171 77 Stockholm, Sweden; 2 Center for Epidemiology and Community Medicine, Stockholm County Council, 171 29 Stockholm, Sweden; 3 Department of Medical and Health Sciences, National Centre for Work and Rehabilitation, Linköping University, 581 83 Linköping, Sweden

**Keywords:** Local politicians, Community, Policy, Evidence-based practice, Social care

## Abstract

**Background:**

Despite national policy recommending evidence-based practice (EBP), its application in social care has been limited. While local politicians can affect the process, little is known about their knowledge, attitudes and roles regarding EBP. The aim here is twofold: to explore the role of local politicians in the implementation of EBP in social care from both their own and a management perspective; and to examine factors politicians perceive as affecting their decisions and actions concerning the implementation of EBP policy.

**Methods:**

Local politicians (N = 13) and managers (N = 22) in social care were interviewed. Qualitative thematic analysis with both inductive and deductive codes was used.

**Results:**

Politicians were rather uninformed regarding EBP and national policy. The factors limiting their actions were, beside the lack of awareness, lack of ability to question existing working methods, and a need for support in the steering of EBP. Thus, personal interest played a significant part in what role the politicians assumed. This resulted in some politicians taking a more active role in steering EBP while others were not involved. From the managers’ perspective, a more active steering by politicians was desired. Setting budget and objectives, as well as active follow-up of work processes and outcomes, were identified as means to affect the implementation of EBP. However, the politicians seemed unaware of the facilitating effects of these actions.

**Conclusions:**

Local politicians had a possibility to facilitate the implementation of EBP, but their role was unclear. Personal interest played a big part in determining what role was taken. The results imply that social care politicians might need support in the development of their steering of EBP. Moving the responsibility for EBP facilitation upwards in the political structure could be an important step in developing EBP in social care.

## Background

Despite a growing number of effective interventions within the field of social care [1, 2], only 20–25% of new programmes are evidence-based [2, 3]. Hence, there is an underutilization of research evidence among social care professionals [4–8], and the use of non-evidence-based methods dominates practice [9, 10]. Evidence-based practice (EBP) emerged as a way of bridging this gap between research and practice [11]. EBP is a decision-making process whereby research evidence, client preferences, and one’s own professional experience are weighted to make decisions concerning treatment [12]. In many countries the interest in EBP in social care has increased, leading to policies emphasizing that practice needs to be underpinned by research evidence [3]. Sweden is no exception; a national policy on EBP was published in 2010. The policy has characteristics of a so-called “soft law” [13]; i.e., a recommendation but not legally binding. The overall goal of the policy is that all social care should work in accordance with EBP. From national level, specific support in terms of regional and local change facilitators are provided in order to achieve the goals of the policy. This illustrates the government’s prioritization of EBP in social care. The change facilitators are also intended to function as intermediators in the dialogue between the national and the local level, i.e. the social care organizations. Despite the support provided for the policy implementation, no specific expectations are stated in the policy regarding what local actors such as politicians, social care management or social care workers should do to reach the goal of the policy. The policy has been criticized for being overly vague, leaving social care organizations without guidance in what to implement and how to go about it [14]. A national evaluation of the policy concluded that its aim has not been achieved, and that social care organizations in Sweden cannot currently be regarded as systematically working in accordance with EBP [15].

The Swedish system for social care, similar to other parts of the public sector, has seen a prominent trend of decentralization in the last decades, with increasing local self-government [16]. A consequence of this has been that local policy making has been increasingly delegated to professionals and managers rather than the local politicians. The influence from non-political councils such as service users has also grown [16]. At the same time, social care organizations have been strongly influenced by new public management, including principles such as management-by-objectives (MBO) and the outsourcing of public services to private organizations, requiring a new way of steering from the administrative as well as the political leadership [17].

Local authorities, i.e. municipalities, are responsible for social care and are regulated by the Local Government Act. Social care encompasses functions such as child welfare, mental health care, drug abuse treatment, financial aid, elderly care, and disability care. The municipalities have great autonomy in making decisions regarding how care is organized. Each municipality is ruled by a municipal council of democratically elected politicians. The municipal council organizes its work through a number of committees. Social care often belong to the social welfare committee [18].

What comes to implementation of EBP in Sweden and internationally, the success in the implementation has shown to be affected by factors on several levels, from individual professionals to policy level [19–23]. However, few studies have investigated the higher hierarchical levels of the system, such as the policy level, in relation to EBP. This is surprising, as practitioners who are expected to work in accordance with EBP are affected by factors on these higher levels. Politicians shape the environmental conditions that influence practitioners’ behaviour [24, 25]. More specifically, local politicians at the community level influence how the local social care organizations implement national policy. For example, they can make decisions that affect what types of services are provided and what resources are available [26]. Thus, local politicians’ decisions may either hinder or facilitate the practical implementation of EBP. Political support and funding stability have been associated with the sustainment of EBP and prevention programmes in community settings [27, 28]. In a study by Kalkan et al. [29], political decisions on a regional level influenced health care professionals’ behaviours concerning the prescription of an evidence-based treatment. In another study, political attention affected the degree to which social care workers acted in line with national policy concerning unemployment [30].

When it comes to research use in general among decision makers in health services it has been found that research is used as one of many other sources when making decisions [31, 32]. Furthermore, a personal two-way communication with researchers and research that is timely, relevant and summarized are reported as facilitative factors for research use among policymakers [33]. Barriers to research use include poor access to research evidence, poor skills to assess research and negative perceptions about available research, such as lack of relevance in practice [31, 33]. However, not much is known about local politicians’ knowledge and attitudes towards EBP, where research use is one of three knowledge sources that should be weighted in decision making. Political decisions regarding EBP have been described as a “black box”, requiring further research [34]. Research investigating local politicians’ roles and views on quality improvement in health services has found politicians to be quite passive in these issues. A survey among Swedish local politicians found that the majority were unaware of the existence of central national guidelines within social care. They were also less positive towards the guidelines than were senior managers within social care [35]. Only a minority of the politicians believed national guidelines needed to be supported by local political decisions in order to be implemented. In a similar manner, a subsequent study with managers and politicians showed that the respondents agreed that both managers and politicians are responsible for deciding what care should be offered; but that it was unclear how the actual implementation should be facilitated and by whom [26]. Local politicians have also described being passive regarding decisions on the implementation of national quality registers [36]. Furthermore, social care top-level management might have more power than local politicians in decision-making concerning the development of social care [37]. These studies raise questions concerning the level of responsibility, role and power local politicians have regarding EBP.

The aim of this study is twofold: to explore the role of local politicians in the implementation of EBP in social care from their own perspective as well as management’s; and to examine what factors politicians perceive as affecting their decisions and actions concerning the implementation of EBP policy.

### Theoretical rationale

In the field of policy implementation research, much focus has been on how policies on a national level are put into action [38]. This research has been influenced by two perspectives, bottom-up and top-down. A combination of these two has been advised, and Matland’s ambiguity-conflict model is one attempt at accomplishing this [39]. According to the model, there are four types of implementations depending on the degree of ambiguity and conflict regarding problem formulation and solution: administrative implementation (low policy ambiguity/low policy conflict), political implementation (low policy ambiguity/high policy conflict), experimental implementation (high policy ambiguity/low policy conflict), and symbolic implementation (high policy ambiguity/high policy conflict). According to this categorization, the Swedish national policy on EBP can be viewed as experimental implementation since the policy’s goals and means are vague and there is limited conflict in solutions. In experimental implementation the outcomes of the policy depend strongly on the resources and actors on the local level, and the implemented programme will vary from site to site [39]. The Swedish EBP policy was created on a national level (top-down), but because it is formulated vaguely it is up to local implementers to interpret it (bottom-up). This makes the local politicians’ role in the implementation interesting, since they affect environmental conditions for social care professionals.

According to Lundquist [40], the implementation of a policy entails interaction between a policy-maker and local implementers. The implementation is affected by the local implementers’ understanding, willingness and ability regarding the policy. They need to be aware of and understand the policy’s content, and recognize its intentions. This understanding is affected by how the implementer perceives the policy-makers’ steering activities. Willingness concerns the implementers’ more or less conscious and expressed preferences and their attitude towards the policy. An implementer’s ability can include physical factors such as time, money, personnel, equipment or material. It may also encompass the implementer’s competence and ability to make decisions and affect his/her surroundings [40]. The current study uses Lundquist’s model to examine what affects local politicians’ actions in the implementation of the EBP policy.

## Methods

### Design and setting

The study uses a qualitative approach, whereby interviews were conducted with politicians and managers within the field of social care in Sweden.

### Participants

The study consists of two samples: local politicians on social welfare committees (hereafter called politicians) and managers on different levels within social care (hereafter called managers). Purposeful sampling was applied, with the goal of recruiting participants from municipalities of different sizes, geographical locations, and experience of working in accordance with EBP. Managers were recruited first, and in the municipalities where at least one manager participated, politicians (the chairman and vice-chairman of the social welfare committee) were also invited to participate.

Twenty-two managers (20 women and 2 men) from 14 municipalities were included. Their positions varied: eight were head director of social care, four were head of a department, and ten were head of a unit. A total of 30 politicians from the same 14 municipalities were invited by email to participate. Eight politicians chose not to participate, mainly because they had left their political assignment, and nine could not be reached despite several attempts. The final sample consisted of 13 politicians representing nine municipalities: eight chairmen and five vice-chairmen. The gender distribution was four women and nine men.

### Data collection

Semi-structured interview guides for politicians and managers, respectively, were developed, focusing on the respondents’ understanding of EBP and experiences of being involved in decision-making regarding EBP. Example of questions included are “What role does the social welfare committee have in making decisions regarding the care that is provided?”, “What are your experiences of being involved in the work with EBP in social care?”, and “What would you need in order to facilitate working with EBP?”. The interview guides were tested in four pilot interviews (not used in the analysis), and minor adjustments were made to clarify the questions. Telephone interviews were conducted between August and October 2014 for the managers, and between January and February 2015 for the politicians. The interviews lasted an average of 45 min for the managers and 30 min for the politicians, since the questions for the managers covered more topics. For this study, only the parts of the manager interviews that concerned politicians’ roles are used.

### Data analysis

All interviews were audio recorded and transcribed verbatim, and thematic analysis was used [41]. To address the first part of the aim (role of politicians), inductive codes were applied. This meant that reoccurring themes were identified when the transcripts were read through. For the second part of the aim (what affects decisions and actions), Lundquist’s model [40] was used for generating deductive codes concerning understanding, willingness and capability. The first author analysed all transcripts and the last author analysed two and a comparison was made between the codings. Reoccurring patterns were noted across the inductive and deductive codes and therefore, in the next step, all codes were analysed together. The continued process entailed finding differences and similarities in the politicians’ roles and experience, and to define specific themes from the inductive and deductive codes. The content of the themes was discussed by the first and last authors to increase the trustworthiness of the findings.

## Results

**Fig. 1:**
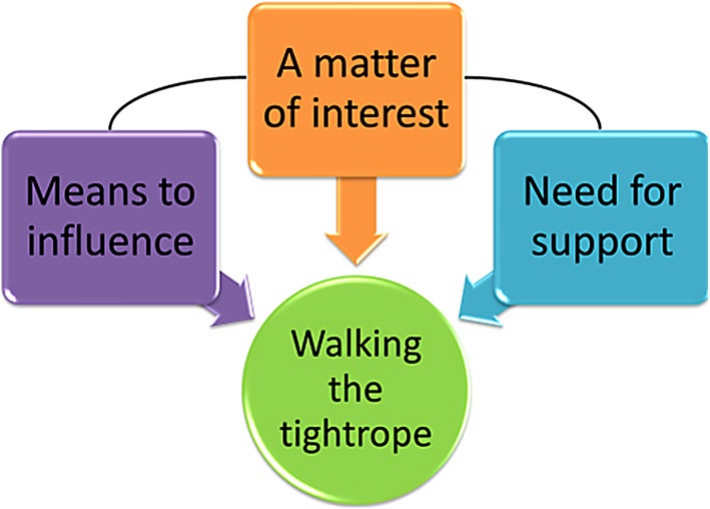
The overall theme, walking the tightrope, and the three sub-themes

An overall theme and three sub-themes were identified (illustrated in Fig. 1). The themes are viewed from both the politicians’ and the managers’ perspectives.

### A matter of interest

#### Politicians

There was little awareness about national policy regarding EBP, and it became clear that there was a breach in the information chain from the national government to local politicians regarding the policy. The picture that emerged was that personal interest was important in determining whether politicians had knowledge about EBP and whether they encouraged it in social care:

*“It must be in the politician’s own interest to acquire the knowledge necessary to make a sensible decision, and it’s up to each individual.” Politician no 9*

The politicians had differing views regarding what role they should have, for instance whether they should make decisions regarding what specific methods are to be used in practice. One perspective was that this was a question strictly for social care professionals and managers:

*“The political committee clarifies what’s to be done and makes resources available for this. That is, the financial and of course personnel resources. The officials//they select the method for solving the tasks.” Politician no 7*

Others suggested that this type of decision should be made by social care management in consultation with the politicians. This was especially true if the method was associated with costs for education or training. Politicians described a fine line between what politicians—as compared to social care professionals and managers—should decide about, something they constantly had to relate to. That decision authority was a challenging matter was illustrated when the politicians’ initially stated that the choice of working methods was a question for social care professionals and managers, but as the interview progressed nevertheless gave examples of situations in which politicians had taken decisions about specific working methods. One politician said (after declaring that they do not make decisions regarding working methods):

*“We decided that when we saw the results. We thought it was so good from a citizen’s perspective//So it was a political decision that we would implement it everywhere.” Politician no 12*

Some politicians took a more active role in steering EBP. They regarded it as an overall approach, which by definition needed to be decided and supported by politicians. These politicians were active through supporting political decisions about EBP, assigning resources, or setting goals that facilitated EBP. They encouraged work with EBP by showing interest in the process. These politicians stressed that they had an important role in making EBP a priority:

*“I think the political committee has a very big task there, to ensure that it (EBP) is under way. And above all that the work is structured, so that one doesn’t only make efforts here and there//and then people change jobs and workplaces, and then suddenly the thing you’ve started is lost. There has to be some kind of continuity to it all.” Politician no 6*

A better understanding of EBP seemed to be related to a more active role in the work with it. However, the politicians rarely had specific knowledge about what EBP entails. It was often referred to as a specific evidence-based method rather than an overall approach to social work. Instead of describing the concept of EBP, most of them talked about recognizing the terminology or remembering having been in contact with it at some point. Furthermore, it was often described in terms of something abstract, ambiguous and difficult to understand.

#### Managers

The managers expressed that political buy-in was essential for successfully working with EBP. This was somewhat contradictory to one of the perspectives among the politicians, that they should not make decisions regarding working methods. Managers wanted the politicians to show interest in, and to distinctly ask for the use of, EBP. Political decisions that facilitated EBP were desired. One of the managers underlined that politicians should demand EBP when deciding on public procurement of services. Others did not know whether politicians had any part in deciding on working methods, since they had little dialogue with the political level. The managers’ responses also mirrored the fact that politicians’ personal interest in EBP played a part in whether or not they actively encouraged it.

*“It’s more about having an interest in the issues and familiarizing yourself with the working methods and the target group, and so forth. There’s no such interest really, but more economic interest.” Manager no 20*

### Means to influence

#### Politicians

The politicians described having great power over three main activities: the provision of resources, setting overall objectives, and following up outcomes. Yet, many of them did not seem to realize that these activities influenced how the work with EBP developed. This was illustrated in their recurrent statements that politicians cannot decide how social care work should be executed. For instance, they did not always consider setting overall objectives to affect what specific methods were used. They were responsible for “what” should be accomplished in the organization, while it was up to the social care professionals to decide “how” this should be accomplished. The politicians described having great influence on the goals that were set for social care and the direction it should take.

Following up concerned getting verbal or written accounts from social care professionals and managers regarding outcomes such as number of clients in treatment and waiting times. Monitoring specific client groups or outcomes on group level was rare. The scope of the follow-up varied from ongoing discussions with social care management to looking at statistics and interviewing clients. It was in the politicians’ hands to initiate the follow-up, and their interests could affect what was requested from social care management. Politicians were aware that they should be more rigorous in their follow-up:

*“To begin with, we’re pretty bad at following up. Secondly, we work with financial monitoring a little too much. We’re not really good at collecting quality assurance. But we try.” Politician no 8*

#### Managers

The managers stressed that politicians could facilitate EBP since its use requires a certain education and training for social care professionals, as well as time and organizational support.

*“You need to be given time and opportunities, and support structures//or get some kind of support in order to be able to benefit from and work extensively with evidence*-*based practice.” Manager no 11*

Many of the managers were in agreement with the politicians regarding the politicians having great power over the overall objectives, but talked more about the need for politicians to be more active in steering towards evidence. Setting overall objectives that harmonize with EBP was seen as a good tool for facilitating it. As regards political follow-up, it became clear that the politicians were not meeting the needs of the social care organizations. It was articulated that following up was an essential part of showing interest in and encouraging the use of certain methods, but that following up was mostly done at the initiative of the managers rather than the politicians.

### Need for support

#### Politicians

There was a general consensus that they need more knowledge about EBP if they are to be able to promote its development. A knowledge and awareness about what EBP entails was described as a postulate for politicians to make it a priority and propel the issue.

*“To have more knowledge about it (EBP), so we can drive this work. I think there are quite a lot of gaps in knowledge//that you don’t know so much about it. I wish we could get more skills and knowledge about this.” Politician no 11*

A reoccurring conception among the politicians was that they did not have sufficient knowledge about EBP (or social work in general) to question the working methods of the social care professionals, or to make decisions or recommendations regarding working methods:

*“I’m not a trained social worker and I don’t have several years of experience behind me; why should I go in and have opinions on their choice of method?” Politician no 5*

The politicians voiced that they need access to relevant and summarized research on social work, presented in a comprehensible way from a layman’s perspective. It was hard for them to get hold of, understand, and judge the quality of research. The politicians often spoke about the value of exchanging experiences with other municipalities regarding social care, getting good examples, and new ideas through workshops and conferences. This was seen as crucial, since research reports seldom gave answers regarding to how to translate research findings into practice.

It became apparent that the politicians were highly dependent on the information they received from the social care professionals and managers. The role of many of the politicians was to be informed about EBP, but not involved. The professionals and managers were pointed out as the primary information source used, since they provided the politicians with not only all the documents to support their decisions but often also information from governmental organizations and state agencies. One politician described it this way:

*“Research and the scientific part is something that’s important when working with decisions and such. That’s where we find the current knowledge//but this is done mainly through the officials.” Politician no 1*

#### Managers

The fact that politicians lacked knowledge about what EBP is arose in the interviews with the managers as well. A manager described politicians’ lack of awareness regarding the needs of the social care professionals in relation to EBP:

*“There’s quite a low awareness about this being a necessary element of our work. So we’ve very little resources to keep us up*-*to*-*date about new developments and research. And also to spend time on following up. It’s something they think we should just have time for.” Manager no 21*

The fact that politicians were dependent on social care professionals and managers for information could pose a problem when the managers wanted politicians to make more demands concerning the use EBP, for example in support documents, since this requires knowledge. Several managers reported having tried to organize information or lectures about EBP and arrange workshops, but that the extent of it all was fairly modest. It was voiced that politicians needed more knowledge about EBP in order to facilitate its implementation, and that a lack of knowledge could be a barrier:

*“The politicians can actually complicate it too. If one isn’t aware that knowledge development, evidence*-*based practice is actually something that’s good for the agency. If you don’t understand that as a politician//then maybe you don’t have an understanding of the officials when they’re presenting different methods and different ways of working. You might want quicker results than what’s delivered and costs, and so forth.” Manager no 20*

### Overall theme: walking the tightrope

*Walking the tightrope* refers to the balance act used to describe the politicians’ role as leading social care without directing too much. The respondents (within both groups) voiced different opinions regarding what role politicians should have, and what they should decide about. The differing opinions also concerned the extent to which politicians should be involved in the performance and development of social care. Some argued that developmental issues were a question for the social care management, while others believed politicians have the responsibility to develop the quality in social care. The politicians described a balance act whereby they were dependent on social care professionals and managers for knowledge and expertise, while at the same time having the duty to assess the quality of work in social care. They also balanced on a tightrope, being laymen as democratically elected politicians while also experiencing a need for more knowledge about EBP in order to better understand and be able to facilitate its implementation of EBP.

## Discussion

We explored what role local social care politicians have in the implementation of EBP, and what factors affect their decisions and actions. We illuminate the politicians’ and social care managers’ perspectives, using Lundquist’s model [40] saying that policy implementation is affected by the local implementers’ understanding, willingness and ability regarding the policy. Our results show that knowledge among the politicians about EBP and the national policy for EBP was low. This was also noted by the managers, who said the politicians did not know enough about the needs of the social care professionals working with EBP. Similar results have been obtained in previous studies, showing that politicians were often unaware of central guidelines in the area of social care [35]. Using Lundquist’s model [40], one can say that the politicians did not fully understand what EBP was and seemed unaware of the national policy, which limited their ability to lead the implementation of EBP. A common conception was that, as politicians, they did not have the expertise required to question the working methods in social care. Furthermore, some of the politicians also lacked willingness; they did not want to assume the role of decision-maker regarding specific working methods. Thus, all three factors in Lundquist’s model affected the politicians’ decisions and actions. A common discussion within municipal systems is to what extent local politicians should steer service provision [16, 17]. In our study, many of the politicians stated that their task is to decide on what is to be done and social care professionals are to decide on how this aim is best reached. Management by objectives (MBO) was thereby an important tool for the politicians. Working with MBO, though, can make the politicians’ role quite contradictory [17]. Local politicians have two roles; one as a democratic representative for the citizens and one as a director of a service-providing system, where they are responsible for the economy and efficiency of the social care provided [17], with decreasing resources at their disposal [16]. They are expected to show concrete results of their work. This can be challenge when one is not directly involved in the development and follow-up of quality issues in social care.

The politicians had different views regarding what role they should have in making decisions about working methods, and specifically EBP. The implication of this was that personal interest determined the role they assumed. Prior studies on politicians have also found ambiguities regarding who on the leading level should facilitate implementation, and how this should be done [26]. Local politicians have been described as passive in decisions regarding the implementation of quality improvement initiatives [36]. It has previously been suggested that there is a “problem of many hands” in complex organizations, making it difficult to determine who is actually accountable for the implementation of research knowledge [42]. This seems to also be the case in the organizations in our study. The managers desired a more active, steering role from the politicians. They wished politicians would be more involved in making decisions regarding EBP, securing resources for education and training, setting goals facilitating EBP, and following up on the quality of care. Both managers and politicians mentioned setting a budget, setting overall objectives, and following up on the work and outcomes as means for steering social care. However, the politicians seemed rather unaware of the facilitating effects of these actions in implementing EBP.

Overall, the steering the politicians exercised in relation to EBP was unconscious and inconsistent. They were making decisions about social care without understanding that this affected the opportunities to implement EBP. Sweden has a national policy for EBP although the goals and means of the policy are vague. In this type of case, the outcomes of the policy depend strongly on the resources and actors on the local level, and the implemented programme will vary from site to site [39]. This is the situation that was found in the current study—politicians’ role in leading EBP implementation was highly dependent on, among other things, their personal interests. Great variations existed among the social care organizations regarding what local politicians (and other local actors) did in the implementation of EBP and how it was understood. Another study has illuminated how vague policy formulation on a national level led to the drifting of an evidence-based method on local levels [43]. Similar findings were also reported by Kalkan et al. [44], where ambiguous national guidelines led to avoidance on a political level. In that study the politicians saw the implementation of the guidelines as an issue for clinical management, which is in line with our results. In both studies, management expressed being dependent on politicians to make political prioritizations in order to facilitate implementation of the policy. The findings illustrate the difficulties national policies can have in influencing social care practices. The question is what kind of policies can be more effective in guiding the local politicians than this type of soft-law policy that leave a great amount of decisions to individual politicians. Policies with clearer goals and steering mechanisms could offer stronger guiding of collective actions and leave less opportunity for individual politicians’ preferences. However, in the Swedish context, this type of policies would probably be experienced as limiting too much the local authorities autonomy, which could in turn evoke negative reactions and probably even unwanted consequences.

The politicians seemed to walk the tightrope when it came to leading the development of social care and EBP. On the one hand they were responsible for the quality of social care, while on the other they were highly dependent on the social care professionals as experts in the field. The politicians often used the professionals as their sole, or predominant, information source when making decisions regarding social care, which could complicate their ability to review the quality of care. It was also often unclear what role politicians were expected to take in relation to the social care professionals. It was common among the politicians to be informed about working methods such as EBP without being involved in the practical work. Their need for support in issues related to EBP and their limited understanding of EBP seemed to affect the actions they took. Politicians with a better understanding of EBP voiced less need for support, and played a more active role in the steering of EBP. The fact that most of local politicians are laymen who fulfil their political assignment in their spare time might complicate the role of local politicians as decision-makers. In light of this, one can discuss to what extent local politicians can be expected to be knowledgeable about EBP or other approaches to social care. Since our results show that the managers require more knowledge about, and steering of, EBP from local politicians, an important issue for governmental organizations should be facilitating this knowledge and steering among local politicians. In line with this, the politicians requested better access to relevant, summarized and comprehensible research on social work. This need is in accordance with research that has found the lack of access to relevant research constitutes a barrier for research use [31, 33]. The scientific community also has a role to play in this, since collaborations between decision-makers and researchers also facilitate the use of research [33].

### Practical implications

It seems that local politicians need support if they are to facilitate the implementation of EBP in social care. Their knowledge, understanding, willingness and ability in steering EBP in social care were limiting effective implementation of the national EBP policy. This resulted in politicians’ personal interest having great impact on what role they took in the process. It is possible that policies with clearer goals and steering mechanisms might be needed if implementation of EBP is a national priority in social care. Stronger guidance of collective actions would leave less opportunity for individual politicians’ preferences. Political attention could be an important key in the further development of EBP in social care, but facilitating EBP seems to be the responsibility of the individual politician. This is a problem since our results show that personal interest was important in determining what role was taken by the politicians. An important change could be to move this responsibility from the individual upwards in the political structure. Municipal councils is the most important decision-making authority in local government and decides on all major matters in the municipalities [16]. If municipal councils were to take decisions regarding adoption of EBP policy, this would lead to politicians and managers in social care being stimulated to facilitate and following up on EBP in social care.

### Methodological considerations

Many politicians had varied—and little—knowledge about EBP. Thus, they may have been referring to different things. Yet, this could have led to a fuller picture of the needs of local politicians. The qualitative nature of the study limits the generalizability of the findings. However, the use of interviews allowed us to explore research questions that were rather unknown, which would have been difficult with a quantitative approach. We analysed the material both inductively and deductively, increasing the richness of the analysis. The use of a theoretical framework enables a theoretical generalizability. Another strength of the study is its use of two respondent groups, which enables a contrasting between the groups. The credibility and trustworthiness were increased by two researchers being involved in the analysis.

## Conclusions

Local politicians have the possibility to facilitate the implementation of EBP in social care. There are differing views on what role politicians should have in this process; if they are to be a part of the implementation, their role needs to be made clearer. Social care managers desired clearer steering from the politicians. The factors limiting politicians’ actions were a lack of awareness of EBP and the policy, ability to question existing working methods, and a need for support regarding the steering of EBP. Personal interest played a large part in what role the politicians assumed. The results imply that social care politicians might need support in the development of their steering of EBP. Moving the responsibility for EBP facilitation upwards in the political structure could be an important step in developing EBP in social care.
