# Correction to: Walking the tightrope-perspectives on local politicians’ role in implementing a national social care policy on evidence-based practice

**DOI:** 10.1186/s13033-021-00471-1

**Published:** 2021-05-12

**Authors:** A. Bäck, C. Ståhl, U. von Thiele Schwarz, A. Richter, H. Hasson

**Affiliations:** 1 Department of Learning, Informatics, Management and Ethics, Medical Management Centre, Karolinska Institutet, 171 77 Stockholm, Sweden; 2 Center for Epidemiology and Community Medicine, Stockholm County Council, 171 29 Stockholm, Sweden; 3 Department of Medical and Health Sciences, National Centre for Work and Rehabilitation, Linköping University, 58 183 Linköping, Sweden

## Correction to: Int J Ment Health Syst (2016) 10:75 https://doi.org/10.1186/s13033-016–0107-1

In the original version of this article [1] one of the quotations was unfortunately misattributed. The first quotation “It’s more about having an interest in the issues and familiarizing yourself with the working methods and the target group, and so forth. There’s no such interest really, but more economic interest” on page 5 should be attributed to manager no 8, not manager no 20 as stated in the original version.
